# Differential amino acid transporter expression in adult *Drosophila melanogaster* tissues

**DOI:** 10.17912/micropub.biology.001121

**Published:** 2024-02-27

**Authors:** Ymani Wright, Alissa R Armstrong

**Affiliations:** 1 Biological Sciences, University of South Carolina, Columbia, South Carolina, United States

## Abstract

Organismal macronutrient intake modulates organ and tissue function. Dietary amino acids play essential roles in metabolic processes that support normal tissue growth, repair, and function. For example, in *Drosophila melanogaster* , protein-deficient diets lead to reduced overall organismal growth during larval development and severely decreased egg production in adult females. Multiple tissues, therefore, must sense and respond to dietary protein input. Amino acid transporter proteins facilitate the movement of amino acids across cellular membranes. Based on high-throughput expression studies, the *Drosophila* genome is predicted to encode 58 amino acid transporters. We have set out to determine if there are tissue-specific amino acid requirements for proper tissue function by first assessing the complement of amino acid transporters expressed in several adult tissues. Using RT-PCR to assess transcript levels, we find that most of the 24 amino acid transporters examined are expressed in the head, thorax, abdomen, gut, and ovary, while a subset shows differential transcript expression. This work will serve as the foundation for future studies addressing the impact of physiological factors, like nutrition, on amino acid sensing by individual tissues.

**Figure 1. Amino acid transporter transcript expression in D. melanogaster tissues:**
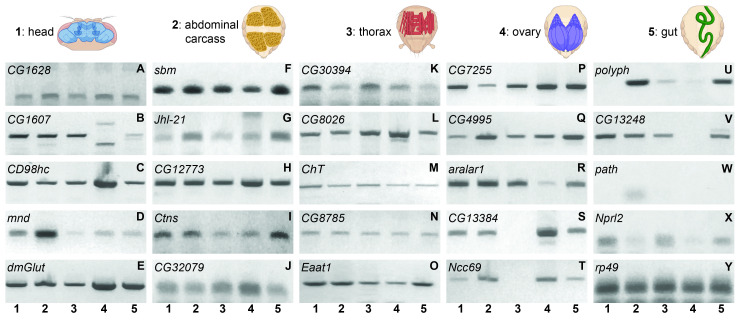
Samples for RT-PCR analyses were taken from five adult tissues, the head (1), abdominal carcass (2), thorax (3), ovary (4), and gut (5). Representative gels are shown for 24 amino acid transporters (A-X) and *rp49* , a loading control, (Y). Tissue order is indicated by numbers at the bottom of each group of gels. Figure created in part with BioRender.com.

## Description

Amino acid transporters (AATs) allow amino acids to move across cellular membranes; thus, modulating amino acid concentration gradients and biochemical pathways (Judge and Dodd, 2020) . For example, AAT activity in pancreatic cell lines can amplify insulin secretion in response to glucose (Bröer, 2022; Javed and Fairweather, 2019). AATs provide cells with amino acids allowing proper tissue-specific function, such as gluconeogenesis in liver cells (Bröer, 2022) and oocyte growth and maturation in mouse ovaries (Pelland et al., 2009) . In *Drosophila* , AATs such as *minidiscs* are critical in growth promotion during larval development (Manière et al., 2020). In adult flies, dietary protein is a major regulator of organismal physiology. In particular, female flies fed a protein-poor diet severely reduce egg production (Drummond-Barbosa and Spradling, 2001) and this is mediated, in part, by adipocyte amino acid sensing (Armstrong et al., 2014) . According to FlyBase, there are currently 58 genes predicted to encode AATs in *Drosophila melanogaster* . While functions for only a few, like *minidiscs* ( *mnd* ), *slimfast* ( *slif* ), and *CD98 heavy chain* ( *CD98hc* ), have been characterized, high-throughput analysis studies suggest that there is differential expression of AATs across tissues (Li et al., 2022; Ceder and Fredriksson, 2022) . Our goal is to reveal the specific amino acid requirements for proper tissue function. As a first step toward this goal, here we describe which AATs are expressed in distinct tissues.

Using RT-PCR, we characterized transcript expression for 24 AATs in the head, thorax, abdominal carcass, gut, and ovary (primer sequences are available for download in the Extended Data section). We find that several amino acid transporter transcripts, *CG1628* , *CG1607* , *CD98hc1* , *mnd* , *Dietary and metabolic glutamate transporter* ( *dmGlut* ), *sobremesa* *(sbm* ), *Juvenile hormone Inducible-2* 1 ( *Jhl-21)* , *CG12773* , *Cystinosin* ( *Ctns* ), *CG32079* , *CG30394* , *CG8026* , *Choline transporter* ( *ChT* ), *CG8785* , *Excitatory amino acid transporter 1* ( *Eaat1* ), *CG7255* , *CG4995* , and a *ralar1,* are detected in all tissues assessed ( Fig. 1A -R). Their broad tissue expression indicates that these AATs function in cellular processes important for all cell types. For example, *CG8026 and CG1628* are implicated in circadian clock regulation (Rivas et al, 2021) which influences protein expression to regulate the biological clock present in most tissues (Patke et al., 2020) . Circadian clock disruption can cause food intake and metabolism defects, leading to conditions such as obesity in humans (Pickel and Sung, 2020) . Individually, *CG1628* , *sbm* , *CG12773* , *CG32079* , *CG8026* , and *CG8785* showed comparable expression levels across tissues ( Fig. 1A, F, H, J, L, N ). While little is known about their molecular and cellular functions in *Drosophila* , studies of human orthologs may shed light on their roles across multiple tissues. *SLC7A9* , the human *sbm* ortholog, transports cystine and its activity is important for protein folding and response to oxidative stress (Goodyer, 2004) . In flies, *sbm* controls cellular and organismal growth as well as developmental timing (Galagovsky et al., 2018) . Thus, *sbm* expression throughout the adult body ( Fig. 1F ) may indicate that it is a general modulator of cell size homeostasis. *CG32079* and *CG8785* have human orthologs in the *SLC36* family of transmembrane transporters, which have roles in amino acid export from neuronal lysosomes, amino acid uptake in intestinal epithelial cells, and amino acid reabsorption at the plasma membrane (Schiöth et al., 2013). Since these AATs are widely expressed and their orthologs have varied roles, using *Drosophila melanogaster* as a model will provide a more comprehensive understanding of their functions.

Amongst the transporters expressed in all tissues, *CD98hc, dmGlut, Jhl-21, Ctns, CG30394, ChT, Eaat1, CG7255, CG4995, aralar1,* and *Nitrogen permease regulator-like 2* ( *Nprl2* ) showed variable expression levels ( Fig. 1C, E, G, I, K, M, O, P -R, X). *ChT* , a choline transporter, has the highest expression in the head ( Fig. 1M ), which supports the role of choline in acetylcholine biosynthesis and thus olfactory neuron function (Hamid et al., 2021; Hamid et al., 2019) . *Eaat1* , excitatory amino acid transporter 1, shows moderately higher expression in the head and gut relative to the thorax and ovary ( Fig. 1O ). It is a sodium-dependent glutamate transporter (Seal et al., 1998) that is regulated by Notch signaling, a highly conserved pathway with multiple functions in multiple cell types (Stacey et al., 2010; Zhou et al., 2022) . It is required for long-term memory storage and reduces seizure activity in epileptic flies (Marquand et al., 2023; Zhang et al., 2022) . Mutations in human *SLC1A3* and *SLC1A1* , orthologs for *Drosophila* *Eaat1* , are associated with two rare amino acid disorders, Glutamate-Aspartate Transporter Deficiency and Dicarboxylic Aminoaciduria, respectively (Mele et al., 2023) . *dmGlut* has the highest expression in the ovary and gut ( Fig. 1E ). *dmGlut* overexpression causes glutamate dependent megamitochondrial formation, suggesting its importance in glutamate regulation (Shim et al., 2012) . While showing low expression levels across all tissues compared to the other AATs examined, *Nprl2* has higher expression in the head and thorax ( Fig. 1X ). Under amino acid starvation, *Nprl2* inhibits Target of Rapamycin Complex 1, thus protecting cells from nutrient stress (Wei and Lilly, 2014) . Mutations in *Nprl2* cause focal epilepsy in humans and motility defects in *Drosophila* (Ricos et al., 2016; Sun et al., 2021; Wei et al., 2016) , suggesting that *Nprl2* is important in motility and motor neuron maintenance, and corroborated by higher expression levels in the head and thorax.

Several AATs show differential expression in a more restricted pattern. *CG13384* and *sodium chloride cotransporter 69* ( *Ncc69* ) are expressed in all tissues examined except the thorax ( Fig. 1S, T ) *.* Knockdown of *CG13384* blocks salivary gland degradation (Velentzas et al., 2018) , suggesting that this AAT may be important for regulating autophagy and/or apoptosis in the brain, fat body, ovary, and gut. *Ncc69* mutant flies had reduced fluid secretion rates and K+ flux, suggesting that *Ncc69* may be important for fluid and electrolyte homeostasis (Rodan et al., 2012) . Undetectable in the head, *polyph (polyphemus)* shows low expression in the thorax and ovary and high expression in the abdominal carcass and gut ( Fig. 1U ). As part of the immune system, *polyph* promotes phagocytosis of microbes after infection (Gonzalez et al., 2013) , thus confirming its robust expression in the abdominal carcass, which houses the fat body and hemocytes, major mediators of the *Drosophila* immune response. *CG13248* is expressed in all tissues examined but the ovary ( Fig. 1V ). *CG13248* transports histidine in fly photoreceptors (Han et al., 2022) and is also highly expressed in neurons promote food intake in response to dietary amino acids (Yang et al., 2018) , corroborating its highest expression level in the head. We also find that *pathetic* ( *path* ) is only expressed at very low levels in the abdominal carcass of adults ( Fig. 1W ), in contrast to the broad tissue expression observed in larvae (Lin et al., 2015) .

In *Drosophila* , the fat body and ovary are highly nutrient-responsive tissues (Zheng et al., 2016; Armstrong, 2020) . Previous studies demonstrate that conserved nutrient-sensing pathways, like insulin/insulin-like growth factor signaling (IIS), mechanistic Target of rapamycin (mTOR) signaling, and the amino acid response pathway, act within the fat body to modulate the ovarian response to diet (Armstrong et al., 2014; Armstrong and Drummond-Barbosa, 2018) . Transcripts for all AATs examined are detected in the abdominal carcass, which primarily contains the adipose tissue, with 15 showing moderate to high expression levels ( *CG1607, CD98hc, mnd, dmGlut, sbm, CG12773, Ctns, CG8026, Eaat1, CG4995, aralar1, CG13384, Ncc69, polyph,* and *CG13248* ). The ovary expresses all but three AAT transcripts, with 10 showing moderate to high expression levels ( *CD98hc, dmGlut, sbm, CG12773, CG32079, CG8026, Cg7255, CG4995, Cg13384,* and *Ncc69* ). This indicates that AATs transport dietary amino acids into adipocytes and ovarian cells to directly modulate nutrient-sensing pathway activity. In fact, *JhI-21* and *mnd* in insulin-producing cells in the larval brain sense leucine to regulate *Drosophila* insulin-like peptide 2 secretion (Ziegler et al., 2018; Manière et al., 2016). *path* , which transports alanine, glycine, and proline, has been shown to regulate growth via IIS/mTOR signaling (Goberdhan et al., 2005). Moreover, adipocyte-specific knockdown of *CG1607, CG1628, ChT, CG12773* , and *CG13384* results in decreased egg production, in part due to ovarian germline stem cell loss and blocked ovulation of mature oocytes (Armstrong et al., 2014) .

In this study, we show that AAT transcript expression varies in adult wild-type *Drosophila* tissues. While all cells employ biochemical pathways to support proper tissue function, they do so in a context-dependent manner. For instance, compared to other tissues, the energy balance function of adipocytes modulates fatty acid oxidation (Kummitha et al., 2014) . Having a thorough understanding of differential AAT expression will uncover tissue-specific contexts of diet-dependent biochemical pathway activity. For example, dietary enrichment of essential amino acids, particularly leucine, increases AAT protein expression, and thus mTOR signaling, in human skeletal muscle cells (Drummond et al., 2010) . Organismal physiological condition also impacts tissue function **.** Thus, changes in AAT expression in response to diet, age, sex, and other external factors may mediate tissue-specific changes in function. For example, sexually dimorphic morphological features (Rideout et al, 2015; Surkova et al., 2021) , cellular functions (Stowers and Logan, 2010; Belmonte et al., 2019) , and molecular signatures (Jin et al., 2001) underlie sex-specific biological functions like mating behaviors (Manoli et al., 2013) and gonad development (Whitworth et al., 2012; Grmai et al., 2022) . While this study used mixed-sex samples, whole transcriptome sequencing indicates male-biased expression of several AATs, such as *Eaat1, Ncc69, ChT, kcc, CD98hc* , and *CG13384* (Chang et al., 2011). Therefore, future studies, in addition to characterizing the full complement of AATs expressed across tissues and cell types, should determine how physiology influences transcript and protein expression. Furthermore, this information will support functional studies to elucidate the molecular and cellular processes regulated by amino acid sensing.

## Methods

*Drosophila stock and husbandry*

The wild type IV stock, derived from a North American ancestral population established over 40 years ago (Houle and Rowe, 2003) , was maintained at 22-25° C on corn syrup-based medium containing agar, cornmeal, and yeast. Male and female virgins were collected within 24h of eclosion for age matching and were fed a molasses-based medium containing agar, cornmeal, and yeast for five days prior to dissection.

*RT-PCR analysis*

Heads, thoraces, abdominal carcasses, ovaries, and guts were dissected in RNAlater from a total of 20 flies and RNA was extracted using the Zymo Research Quick-RNA Miniprep Kit. The ThermoScientific Verso cDNA synthesis kit was used to generate complementary DNA with anchored oligo dT primers. We used FlyBase (release FB2023_06) to identify AATs encoded by the *Drosophila melanogaster* genome. Using the ‘Gene Ontology’ search tool, AATs were identified with the GO term ‘amino acid transmembrane transporter activity’ (GO:0015171) under molecular function (Gramates et al., 2022). Of note, CG8026 and Nprl2 were not identified in FlyBase release FB2023_06, however, they were identified in a 2015 release. Using Primer-BLAST, primers for each amino acid transporter were generated with the following criteria: primers must span an exon-exon junction, product size between 100 to 500 base pairs, and organism must be *D* . *melanogaster.* Primers used to amplify cDNA for each amino acid transporter are available for download in the Extended Data section. After testing primers for the ability to amplify a product on whole-fly cDNA, a temperature gradient was used to identify optimal annealing temperature. Following PCR, products were run on a 1.5% agarose gel and viewed on an Acure UV transilluminator.

## Reagents

ThermoScientific Verso cDNA synthesis Kit (Catalog # AB1453A)

Invitrogen RNAlater (Catalog # AM7023)

Zymo Research Quick-RNA Miniprep Kit (Catalog # R1054)

Integrated DNA Technologies (Primers)

1% Tris Borate EDTA Buffer

Archon Scientific Molasses Vials (Catalog # B20102)

Biosearch EconoTaq PLUS Green (Catalog # 30033-1)

1X PBS (For dissecting)

New England Biolabs Quick-Load Purple 100 bp DNA Ladder (Catalog # N0551S)

## Extended Data

Description: Table of PCR primers used in this study. Resource Type: Dataset. DOI: 10.22002/q3d5m-mp447
